# Structure‐Guided Design of Peptides as Tools to Probe the Protein–Protein Interaction between Cullin‐2 and Elongin BC Substrate Adaptor in Cullin RING E3 Ubiquitin Ligases

**DOI:** 10.1002/cmdc.201700359

**Published:** 2017-09-01

**Authors:** Teresa A. F. Cardote, Alessio Ciulli

**Affiliations:** ^1^ Biological Chemistry and Drug Discovery Division School of Life Sciences University of Dundee Dow Street DD1 5EH Dundee UK

**Keywords:** chemical probes, Cullin RING E3 ligases, peptides, protein–protein interactions, structure-guided design

## Abstract

Cullin RING E3 ubiquitin ligases (CRLs) are large dynamic multi‐subunit complexes that control the fate of many proteins in cells. CRLs are attractive drug targets for the development of small‐molecule inhibitors and chemical inducers of protein degradation. Herein we describe a structure‐guided biophysical approach to probe the protein–protein interaction (PPI) between the Cullin‐2 scaffold protein and the adaptor subunits Elongin BC within the context of the von Hippel‐Lindau complex (CRL2^VHL^) using peptides. Two peptides were shown to bind at the targeted binding site on Elongin C, named the “EloC site”, with micromolar dissociation constants, providing a starting point for future optimization. Our results suggest ligandability of the EloC binding site to short linear peptides, unveiling the opportunity and challenges to develop small molecules that have the potential to target selectively the Cul2‐adaptor PPI within CRLs.

Cullin RING E3 ubiquitin ligases (CRLs) are key machineries of the ubiquitin proteasome system as they are responsible for catalyzing the final step in the ubiquitination cascade, in which a ubiquitin molecule is transferred to the substrate.^1^ CRLs, of which over 230 are estimated in human cells, are responsible for approximately 20 % of the ubiquitin‐dependent protein turnover in cells, being implicated in a number of cellular processes across different organisms.^2^ The significant roles of CRLs in several biological processes and human diseases has rapidly emerged, in particular in cancer, where the genes encoding for E3 ligase subunits and their native substrates are often found as oncogenes or tumor suppressors.^3^ Currently, much focus is directed toward targeting E3 CRLs with small molecules, such as inhibitors, to block the ligase activity;^4^ molecular glues, to redirect E3 CRL activity toward neo‐substrates;^5^ and bivalent PROTACs, to induce targeted protein degradation.^6^, ^7^ These chemical modalities motivate the growing interest in studying this class of enzymes. While E3 inhibitors, molecular glues and PROTACs have been widely reported, to our knowledge there are only a few examples of small molecules developed to disrupt inter‐subunit assembly within CRLs.^8^, ^9^

Our work focused on probing a particular protein–protein interaction (PPI) in the CRL2^VHL^ ligase. The central scaffold of the VHL ligase is Cullin‐2 (Cul2), which recruits at the N‐terminal domain the von Hippel‐Lindau protein (pVHL) as substrate receptor, through an adaptor subunit constituted by Elongin B (EloB) and Elongin C (EloC) and at the C‐terminal domain the RING box protein, Rbx1 (Figure 1 B). We were interested in an epitope of the PPI between Cul2 and the receptor‐adaptor trimeric subunit composed by pVHL, EloB and EloC (VBC). This PPI has been described as comprising three main points of interaction.^10^, ^11^ The first crystal structure (Ref. 10) comprised VBC bound to the first helical bundle of Cul2_NTD_, whereas a recent crystal structure (Ref. 11) reported by us comprises the whole CRL2^VHL^ complex. The latter work unveiled the importance of hydrophobic residues for the tight binding affinity observed between VBC and Cul2 (*K*_d_=42 nm). In this work, we focused our attention on the contact surface between the N‐terminal tail of Cul2 and EloC, which we refer to as EloC site.

**Figure 1:**
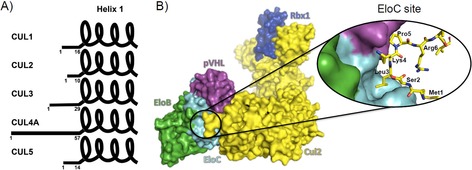
A) N‐terminal tail in the Cullin family. The extension of the N‐terminal tail, residues ahead of the first α‐helix, is variable across the Cullins and plays different roles in the respective PPIs. B) The CRL2^VHL^ complex. Crystal structure of the CRL2^VHL^ complex (PDB ID: 5N4W)^11^ composed by pVHL as receptor subunit, EloB and EloC as adaptor subunit, Cul2 as scaffold subunit, and Rbx1 as RING finger domain. Close‐up view of the PPI between the N‐terminal tail of Cul2 and VBC.

Targeting PPIs with small molecules provides many opportunities for basic biology and molecular therapeutics, but the physicochemical nature of these interfaces turns the ability to modulate them into a great challenge.^12^, ^13^, ^14^ Therefore, the identification and development of binding ligands to protein surfaces, whether direct or allosteric modulators of PPIs, remains a difficult and unsolved problem. Fortunately, much progress has been made in recent years in this direction. In particular, it is becoming evident that the development of drug‐like PPI inhibitors, and small‐molecule ligands to protein surfaces, can greatly benefit from the availability of a peptidic ligand to that binding site, which could be from the natural interacting partner or from synthetic sources.^15^

We hypothesized that Cul2‐derived peptides binding to the EloC site could provide valuable insight on how to target the Cul2‐VBC PPI. Previous work in our research group has led to the development of potent small‐molecule disruptors of the pVHL‐HIF‐1α PPI based on the structure of pVHL bound to a 19‐mer parental peptide derived from HIF‐1α.^16^, ^17^, ^18^ It was therefore an attractive strategy to explore the potential to apply a similar approach to other non‐HIF binding surfaces on VBC. Such peptidic ligands could inform on the nature and details of key interactions essential to achieve affinity at the targeted binding site. They could also provide useful displacement tools to ensure specificity of interaction of compound series in ligand development campaigns. Furthermore, peptides are interesting candidates as PPI modulators themselves, presenting a number of advantages over non‐peptidic small molecules: biocompatibility, and low toxicity to the organism; chemical flexibility, such as the ability to adapt to large and often flexible surfaces; modularity, thus enlarging the structural diversity, enhancing selectivity and leading to high potency.^19^

Considering the important role of the N‐terminal tail of Cul2 in establishing the interaction with VBC (Figure 1), it was hypothesized that short peptides able to reproduce this tail could recapitulate the interaction, providing tools to develop chemical probes and target this PPI. Based on the structural analysis of the Cul2‐VBC interface, we first aimed to recapitulate the interaction using N‐terminal Cul2 peptides varying from three to eleven residues. The peptides were synthesized and seven of the nine peptides were tested for binding to VBC by Biolayer Interferometry (BLI) (the 9‐mer and 11‐mer peptides could not be tested because they formed a white precipitate in the conditions of the experiment). The results showed that at least six amino acids were required to observe binding to VBC. The binding event was recapitulated with the 6‐, 7‐, 8‐ and 10‐mer peptides; nevertheless, the binding affinities determined were quite weak (*K*_d_ in the millimolar range, Figure 2). Thus, the next step was to enhance the binding affinity of the peptides toward VBC.

**Figure 2:**
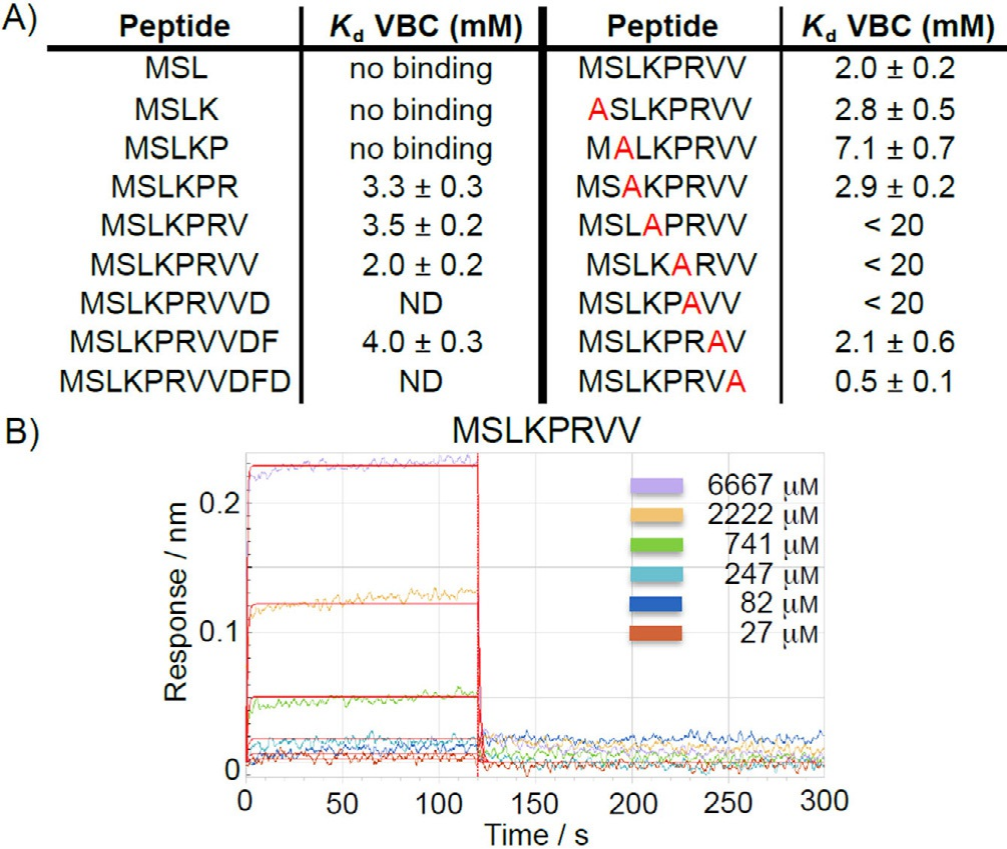
Biolayer interferometry data of N‐terminal Cul2 peptides versus VBC. A) Binding affinities (*K*_d_ values) obtained from the BLI experiments. B) Representative sensorgram of the 8‐mer peptide titrated against VBC.

Considering the 8‐mer peptide (MSLKPRVV) had the best fitting, tighter binding affinity and reproducibility in the previous BLI assay, it was chosen as template for an alanine scanning to identify hotspots in the 8‐mer peptide. By replacing, one at a time, all the amino acid residues by an alanine residue we observed that upon replacement of Pro5 or Arg6, the binding of the peptide toward VBC was totally lost (Figure 2). This was in agreement with the structural analysis of the Cul2‐VBC complex,^10^ which suggests that Pro5 is responsible for the folding of the N‐terminal tail of Cul2 upon itself and Arg6 is responsible for keeping this conformational arrangement by establishing intramolecular hydrogen bonds with the carbonyl groups from the backbone of Ser2 and Lys4. Replacing Lys4 with alanine resulted in a 10‐fold decrease in the binding affinity but the replacement of other amino acids in the peptide with alanine did not seem to perturb the interaction as much. The alanine scanning results also implied that the leucine residue, of which the side chain inserts into the EloC site could be replaced without significant loss of binding affinity.

From the results of the initial screen we learned that: 1) the preferential size of the peptide comprised eight amino acids; 2) Pro5 and Arg6 were critical to assure binding to VBC; and 3) Leu3 could be replaced without major loss of binding affinity. Thus, we employed a structure‐based approach to enhance the affinity of the 8‐mer peptide, by replacing the leucine with amino acids presenting bulkier side chain groups. A small library of 8‐mer peptides containing a set of natural and non‐natural amino acids replacing Leu3 was designed and tested for binding to VBC. The criteria for choosing these amino acids was to modulate the bulkiness of the side chain group, for example, we chose phenylalanine and *tert*‐butylglycine, among others, to increase the volume occupied by the side chain. As before, the peptides were initially tested by BLI for binding to VBC (Table 1). The BLI results showed that the EloC pocket could accommodate all the derivative peptides, except when leucine was replaced by tryptophan, which was probably overly bulky and was found to decrease the binding affinity relative to the parental peptide. In addition, it was also observed that the replacement of leucine with *tert*‐butylglycine and dimethylcysteine (peptides **G** and **J**, respectively) led to the dissociation constant breaking into the micromolar range. Particularly for peptide **J**, the *K*_d_ value determined by BLI was 0.3±0.1 mm, which represents a 6‐fold improvement in regards to the parental peptide **A**. These results encouraged further in‐depth characterization of peptide **J**, with the aim to better understand its binding interaction.

**Table 1:** Results of the BLI experiment with the peptide variants replacing Leu3 (highlighted in the structure below).

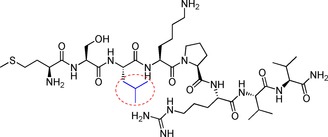			
Peptide	Leu3 replacement		*K*_d_ [mm]
**A**	leucine	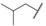	2.0±0.2
**B**	tryptophan	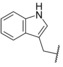	18±1
**C**	phenylalanine	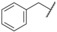	3.1±0.2
**D**	tyrosine	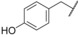	7.4±0.4
**E**	norleucine	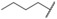	ND^[a]^
**F**	norvaline	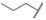	1.9±0.2
**G**	*tert*‐butylglycine	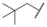	0.9±0.1
**H**	phenylglycine	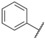	2.1±0.6
**I**	cyclohexhylglycine	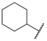	8.9±0.7
**J**	dimethylcysteine	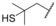	0.3±0.1
**K**	thioproline	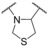	2.2±0.4

[a] The *K*_d_ value of peptide **E** could not be determined because the fitting function was not adequate for the data, suggesting that other effects in addition to genuine and reversible 1:1 binding likely account for the observed signal.

The binding of peptide **J** to VBC was characterized further by other biophysical techniques in addition to BLI, namely isothermal titration calorimetry (ITC), AlphaLISA competition assay, and protein‐observed nuclear magnetic resonance (NMR), which all showed consistent results. Titration of peptide **J** into VBC by ITC resulted in the determination of *K*_d_=5.28×10^−4^±0.65×10^−4^ m (Figure 3 B). This three‐digit micromolar *K*_d_ corroborated the *K*_d_ value obtained by BLI (Figure 3 A). In the AlphaLISA assay, peptides **A** and **J** were used to disrupt the native interaction between VBC and Cul2. Both peptides **A** and **J** were found to displace Cul2 (Figure 3 C).

**Figure 3:**
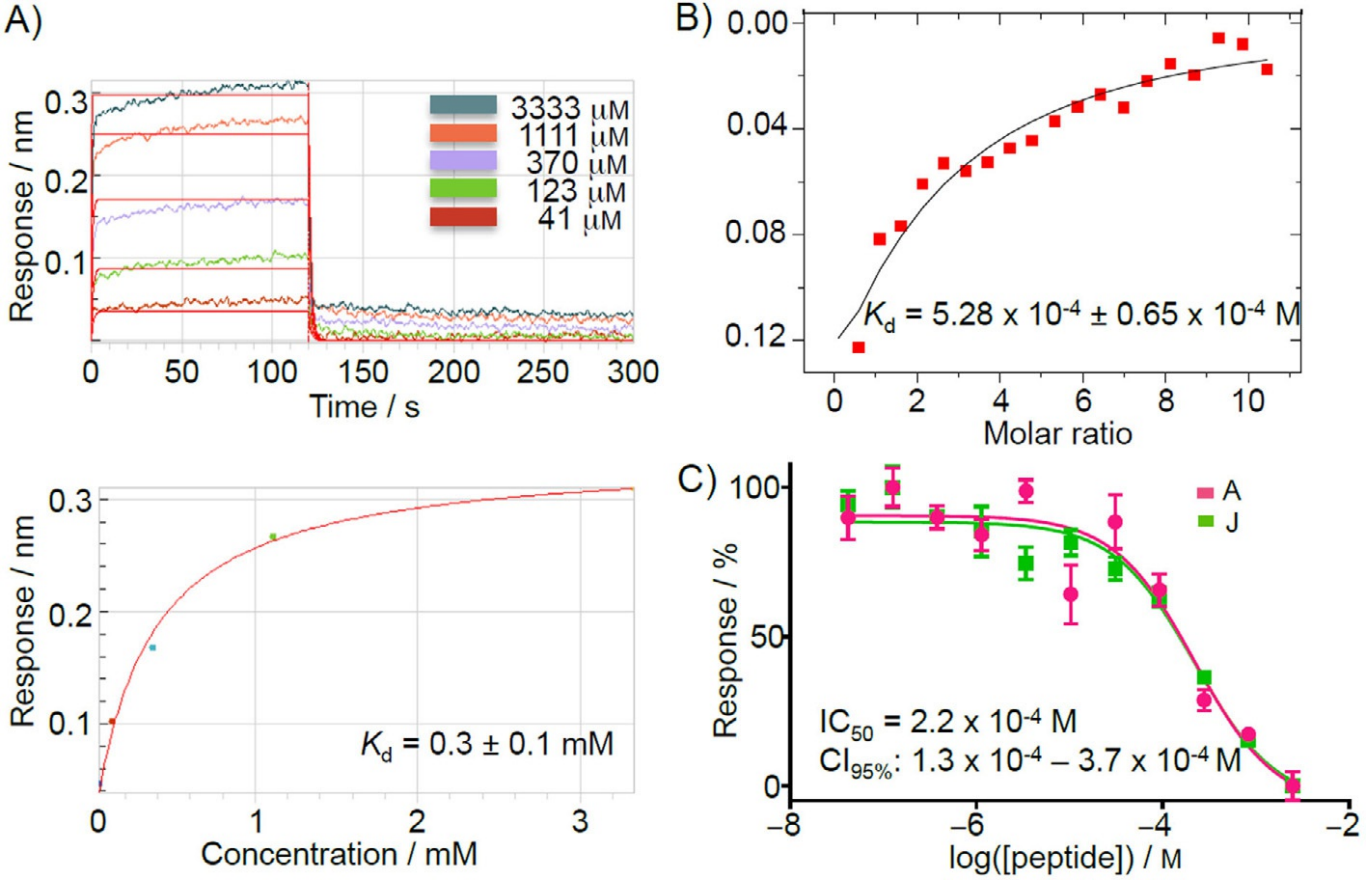
Biophysical characterization of peptide **J** binding to VBC. A) BLI results. Peptide **J** was titrated against VBC and a *K*_d_ value was determined based on the fitting of the response data points. B) ITC experiment. Peptide **J** (5 mm) was titrated against 100 μm VBC at 298 K. C) AlphaLISA results of the titration of peptides **A** and **J** in a competition experiment to displace Cul2.

The IC_50_ determined for peptide **J** in the AlphaLISA displacement assay was 0.22 mm, a value similar to the *K*_d_ values for direct binding measured by ITC and BLI. However, the AlphaLISA assay could not distinguish the binding of peptide **J** from peptide **A**, which had shown 10‐fold weaker binding affinity by BLI, presumably due to differences between the two assays. Nonetheless, the AlphaLISA results clearly validated the binding to the Cul2 binding site. Finally, we performed chemical shift perturbation (CSP) experiments by protein‐observed NMR and the results suggested that the peptide was binding to VBC. Additionally, the data was in agreement with the AlphaLISA, suggesting that peptide **J** was binding to the EloC pocket (Figure 4). Upon binding to a certain area of the protein, the peptide changes the chemical environment of the residues that surround it. These changes in the chemical environment are registered as peak shifting or disappearance. The residues affected by the binding of the peptide were identified based on the peak assignment available for VBC (provided by Dr. Mark Bycroft, Cambridge).^20^ It was remarkable that whilst some peaks were undeniably affected, others remained constant. Mapping the disturbed residues on the structure suggested that the residues more affected by the presence of the peptide were near the EloC pocket (Figure 4 B). There were also some other peaks shifting that correspond to residues in different areas of the protein. It is expected that amino acids at a certain distance from the binding site might rearrange upon binding of a ligand and, hence, promote shifts.

**Figure 4:**
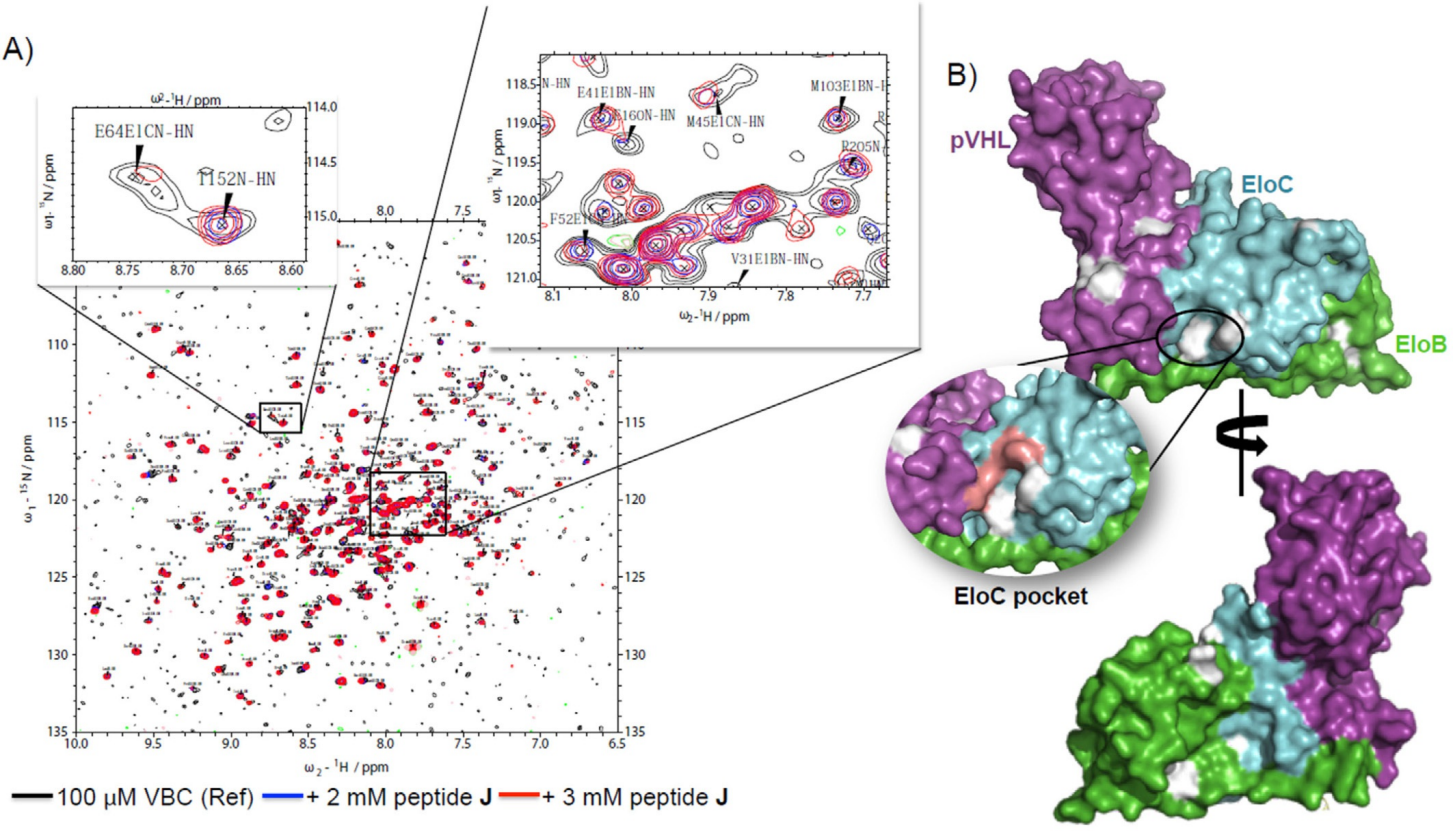
Chemical shift perturbation analysis by NMR spectroscopy. A) HSQC spectra with zoom in regions where it is possible to observe peaks being affected by the binding of peptide **J** and peaks not being affected. B) Mapping of CSP in VBC. Affected residues are shown in grey. Residues in EloC pocket have shifted, suggesting binding of the peptide in the expected binding site. Residues marked in pink could not be identified in our spectra, thus it is not clear whether the chemical shifts from those residues have been perturbed in the presence of the peptide.

The biophysical characterization suggested moderate binding to VBC and it disclosed the opportunity to develop these Cul2 peptides into high‐affinity binders. The strength of the interaction was boosted about 4‐fold simply by increasing the volume of the side‐chain fitting the EloC site. It is anticipated that the structure of Cul2 N‐terminal tail bound to VBC can differ significantly when in the context of an 8‐mer peptide or in the context of the full‐length protein. The structural knowledge of the binding mode of peptide **J** in complex with VBC would help to inform molecular design to enhance binding. Considering this, efforts were taken into co‐crystallizing peptide **J** with VBC, however, to date this has not been achieved.

In conclusion, it has proven challenging to target this particular PPI using peptides. The VBC‐Cul2 interaction appears to be a tertiary PPI,^21^ involving multiple epitopes. In fact, as observed in the crystal structure, the interaction is directed by three points of contact between pVHL, Cul2 and EloC. Despite the N‐terminal tail of Cul2 being important for the specificity of the interaction, by itself it could not drive a tight binding event. Therefore, peptides that target only one of these interaction sites would likely not be able to mimic the native interaction and thus would not block the PPI site effectively. Other drug discovery tools such as peptide stapling,^22^ or tethering,^12^ for example, might be helpful toward this goal. Another approach to target this kind of PPIs could be the use of bicyclic peptides as they cover a large surface area and are able to closely mimic PPI features.^23^

Our work demonstrates how demanding it can be to target extended PPI regions, as opposed to well‐defined binding‐sites. In this particular case, it suggests that the narrow and merely hydrophobic nature of the EloC site make it a challenging target site. The gained knowledge and tools will nevertheless inform future development of small molecules that could target this specific PPI and could be used as chemical probes to study Cul2‐dependent CRLs.

## Experimental Section

**Protein expression**: VBC ternary complex was described previously.^17^ BL21(DE3) *E. coli* cells were co‐transformed with the plasmid for expression of pVHL/SOCS2 and the bi‐cistronic pDUET plasmid for expression of EloBC. A single colony of transformant was used to inoculate LB media for bacterial culture. Protein expression was induced with 0.3 mm IPTG (when OD_600_ reached 0.8) at 24 °C for 18 h. Co‐expression of these proteins resulted in the formation of the respective trimeric complex (VBC) that was then purified by two steps of affinity chromatography, followed by ion‐exchange chromatography and finally by size‐exclusion chromatography. The His‐tag was cleaved between the two affinity chromatography steps with TEV protease. Following this protocol the yield of protein was about 15–20 mg per liter of culture. For the expression of ^2^H,^15^N‐VBC, the LB media was replaced with *E. coli*‐OD2 enriched media (Silantes) and the yield dropped to 4 mg L^−1^.^24^

**Peptide synthesis and purification**: the peptides were synthesized in an INTAVIS RespepSL automated peptide synthesizer using solid phase Fmoc chemistry. The peptides were cleaved from the resin using a solution of TFA, water and triisopropylsilane (TIS) (92.5:2.5:5). The peptides were obtained as C‐terminal amides and were purified by HPLC in basic conditions (0.1 % NH_4_OH) in a gradient of 0–100 % of acetonitrile in water over 15 minutes. The purity and identity of the peptides was determined by LC–MS.

**Biolayer interferometry**: BLI experiments were performed in an Octet RED384 (FòrteBio). Biotinylated VBC (25 μg mL^−1^) was immobilized on Super Streptavidin‐coated biosensor (FòrteBio). The experiments were conducted at 25 °C, in 20 mm HEPES pH 7.6, 100 mm NaCl, 1 mm DTT and 0.02 % (*v*/*v*) Tween‐20 buffer. The response of the reference tips was subtracted from the signal to account for unspecific binding. The data points were fitted using a 1:1 model.

**Isothermal titration calorimetry**: ITC experiments were carried out in an ITC200 microcalorimeter (Malvern). All protein solutions were dialyzed into 100 mm Bis‐tris propane pH 8.0, 50 mm NaCl, 2 mm TCEP prior to the titrations. The peptide (5 mm) was titrated into VBC (100 μm). The titrations consisted of 19 injections of 2 μL each (120 sec spacing and 600 rpm stirring speed) at 30 °C.

**AlphaLISA**: Anti‐His_6_ acceptor beads and Streptavidin donor beads (PerkinElmer) were used. The competition assay was performed in a 384‐well plate by mixing V_6×His_BC (500 nm) and biotinylated Rbx1‐Cul2 (150 nm) and titrating the competitor (peptide). The final volume of each well was 20 μL. The plate was then read in a PHERAstar FS (BMG LABTECH). Each of the competitors was titrated in quadruplicate. The fitting and IC_50_ determination were performed in GraphPad Prism 7 (GraphPad Software, La Jolla, CA, USA).

**NMR spectroscopy**: NMR experiments were carried out in an AV‐500 MHz Bruker spectrometer equipped with a 5 mm CTPXI ^1^H‐^13^C/^15^N/D Z‐GRD cryoprobe. The total volume of the sample was 200 μL and the experiments were performed in a capillary tube containing 100 μm ^2^H,^15^N‐VBC samples in a buffer of 20 mm KH_2_PO_4_ pH 7.0, 50 mm KCl, 1 mm DTT, 0.02 % NaN_3_ and 15 % of D_2_O. The 2D ^1^H, ^15^N‐HSQC‐TROSY spectra (in the presence or absence of peptide) were recorded with 32 scans and acquisition times of 200 ms for ^1^H and 100 ms for ^15^N, at 30 °C. The spectra were analyzed in CCP NMR^25^ and the chemical shift perturbation (CSP) were calculated according to the following equation: $CSP=\sqrt{\Delta H^{2}+{\left(\Delta N\times 0.14\right)}^{2}}$ , where Δ*H* is the change in proton chemical shift, Δ*N* is the change in nitrogen chemical shift and 0.14 is a scaling factor required to account for the difference in the range of amide proton and amide nitrogen chemical shifts.^26^ A CSP was considered when it was greater than $\bar{x}+2\sigma$ . The backbone assignment of VBC has been made available as by Dr. Mark Bycroft (Laboratory of Molecular Biology, MRC, Cambridge, UK) and shared as a gift.

**Supporting Information**: The raw BLI data are provided in the Supporting Information.

## Conflict of interest

*The authors declare no conflict of interest*.

## Supporting information

As a service to our authors and readers, this journal provides supporting information supplied by the authors. Such materials are peer reviewed and may be re‐organized for online delivery, but are not copy‐edited or typeset. Technical support issues arising from supporting information (other than missing files) should be addressed to the authors.

Supplementary
