# Supplementary material for: Structure‐Guided Design of Peptides as Tools to Probe the Protein–Protein Interaction between Cullin‐2 and Elongin BC Substrate Adaptor in Cullin RING E3 Ubiquitin Ligases
Source: ChemMedChem. 2017 Sep 1;12(18):1491–6. doi: 10.1002/cmdc.201700359 (PMC5639367; doi:10.1002/cmdc.201700359)
Supplement: Supplementary file 1 — Supplementary [file CMDC-12-1491-s001.pdf]

CHEMMEDCHEM
Chemistry & Drug Discovery

Supporting Information

Structure-Guided Design of Peptides as Tools to Probe the Protein–Protein Interaction between Cullin-2 and Elongin BC Substrate Adaptor in Cullin RING E3 Ubiquitin Ligases

Teresa A. F. Cardote and Alessio Ciulli*[a]

cmdc_201700359_sm_miscellaneous_information.pdf

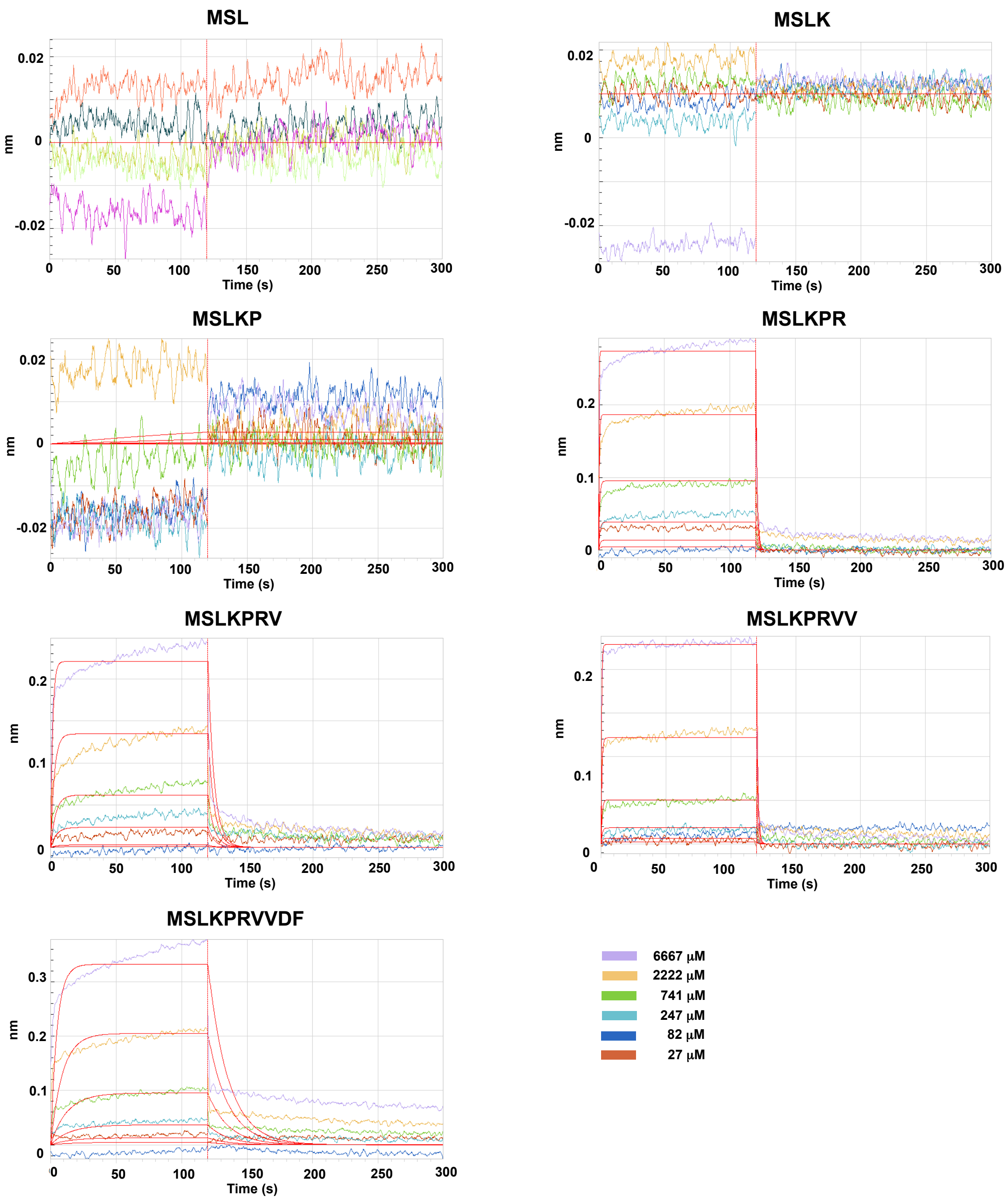

Figure S1 – Biolayer interferometry experiments with the N-terminal Cul2 peptides. Biotinylated VBC was immobilised on the tips and the peptides were tested in a concentration range from 27 μM to 7 mM. The different colours represent the different concentrations and corresponding responses. The assay was performed in 20 mM HEPES pH 7.6, 100 mM NaCl, 1 mM DTT, 0.02% Tween-20 at 25 °C.

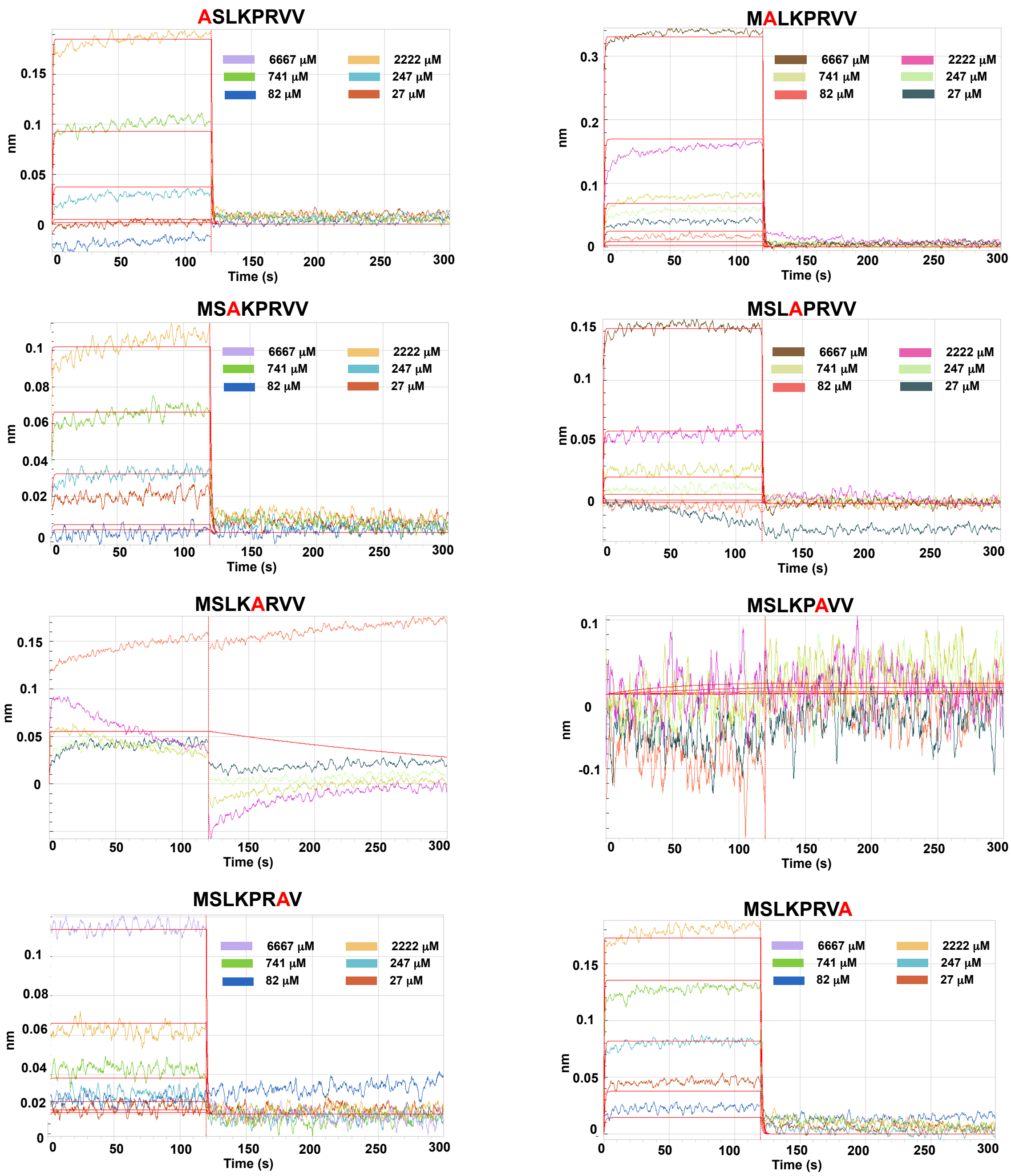

Figure S2 – Biolayer interferomery experiments with the alanine scan peptides. Biotinylated VBC was immobilised on the tips and the peptides were tested in a concentration range from 27 µM to 7 mM. The different colours represent the different concentrations and corresponding responses. The assay was performed in 20 mM HEPES pH 7.6, 100 mM NaCl, 1 mM DTT, 0.02% Tween-20 at 25 °C.

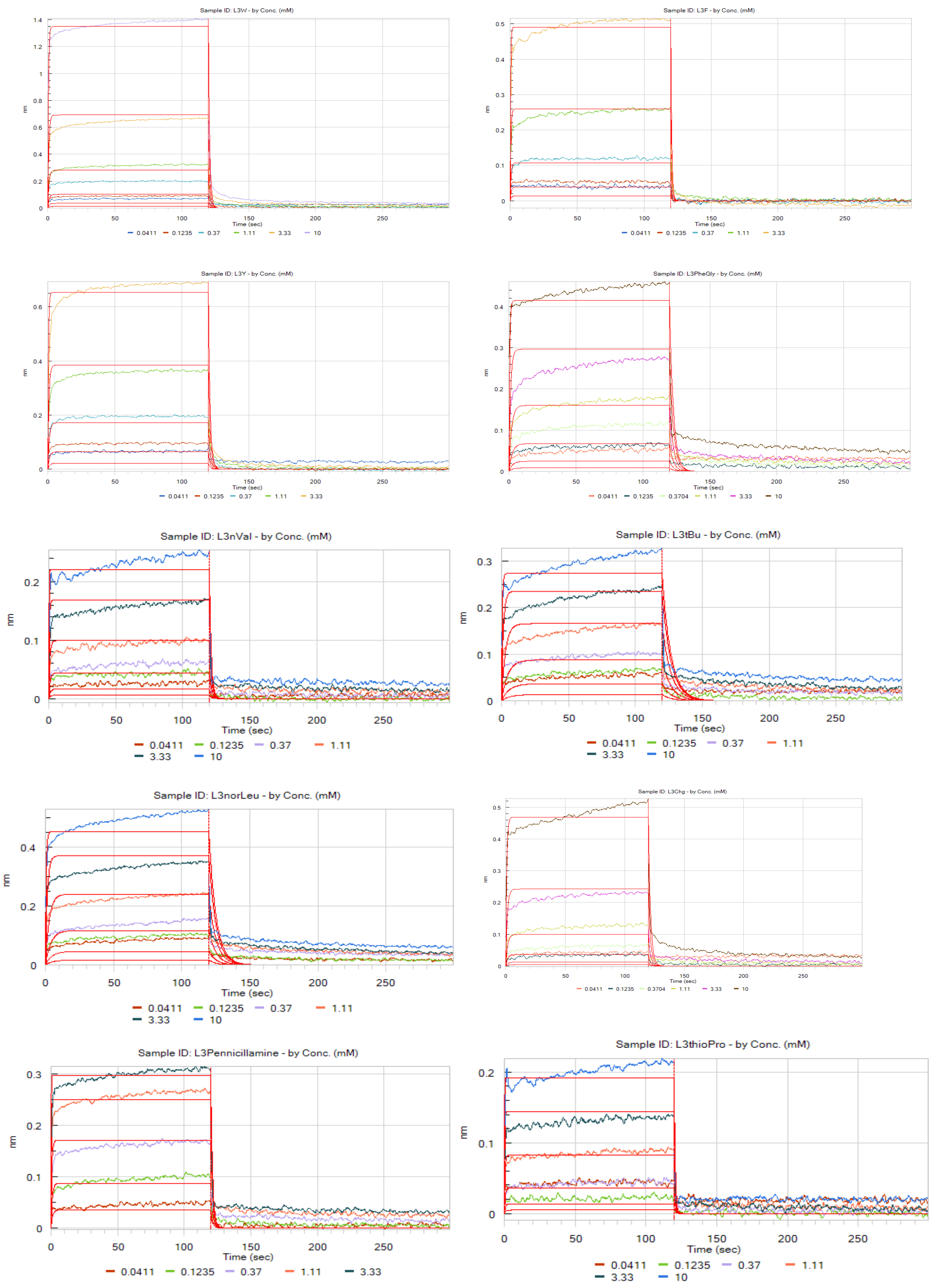

Figure S3 – Biolayer Interferometry data of 8-mer N-terminal Cul2 peptides with leucine replacement *versus* VBC tested on a concentration-dependent assay. Biotinylated VBC was immobilised on the tips and the peptides were tested in a concentration range from 27 μM to 7 mM. The different colours represent the different concentrations and corresponding responses. The assay was performed in 20 mM HEPES pH 7.6, 100 mM NaCl, 1 mM DTT, 0.02% Tween-20 at 25 °C.
